# Germline and somatic mutations of homologous recombination-associated genes in Japanese ovarian cancer patients

**DOI:** 10.1038/s41598-019-54116-y

**Published:** 2019-11-28

**Authors:** Kentaro Sugino, Ryo Tamura, Hirofumi Nakaoka, Nozomi Yachida, Manako Yamaguchi, Yutaro Mori, Kaoru Yamawaki, Kazuaki Suda, Tatsuya Ishiguro, Sosuke Adachi, Masanori Isobe, Masayuki Yamaguchi, Katsunori Kashima, Teiichi Motoyama, Ituro Inoue, Kosuke Yoshihara, Takayuki Enomoto

**Affiliations:** 1 Department of Obstetrics and Gynecology, Niigata University Graduate School of Medical and Dental Sciences, Niigata, 951-8510 Japan; 2 Human Genetics Laboratory, National Institute of Genetics, Mishima, 411-8540 Japan; 3 Department of Molecular and Diagnostic Pathology, Niigata University Graduate School of Medical and Dental Sciences, Niigata, 951-8510 Japan

**Keywords:** Ovarian cancer, Tumour biomarkers

## Abstract

We explored the frequency of germline and somatic mutations in homologous recombination (HR)-associated genes in major histological types of ovarian cancer. We performed targeted sequencing to assess germline and somatic mutations of 16 HR-associated genes and 4 mismatch repair (MMR) genes among 207 ovarian cancer patients (50 high-grade serous carcinomas (HGSC), 99 clear cell carcinomas (CCC), 39 endometrioid carcinomas (EC), 13 mucinous carcinomas (MC), and 6 low-grade serous carcinomas (LGSC)). Germline or somatic mutations of HR-associated genes were detected in 44% of HGSC, 28% of CCC, 23% of EC, 16% of MC, and 17% of LGSC patients. The profile of HR-associated gene mutations was remarkably different among each histological type. Germline *BRCA1/2* mutations were frequently detected in HGSC and were rarely observed in CCC, EC, and MC patients. *ATM* somatic mutation was more frequently detected in CCC (9%) and EC patients (18%) than in HGSC patients (4%). There was a positive correlation between MMR gene mutations and HR-associated gene mutations (p = 0.0072). Our findings might be useful in selection of ovarian cancer patients that should be treated with PARP inhibitors.

## Introduction

Recently, the prevalence of homologous recombination (HR)-associated gene mutations among many tumor types has been characterized^1,2^. In particular, HR pathway alterations are most frequently observed in high-grade serous ovarian carcinoma (HGSC) and breast cancer^2^. It is well-known that around half of HGSC patients exhibit HR deficiency^3,4^. HR deficiency is also associated with response to platinum-based chemotherapies in patients with ovarian cancer^5^, and germline *BRCA1* and *BRCA2* mutations, which are representative alterations causing HR deficiency, are undoubtedly associated with improved prognosis in advanced-stage ovarian cancers^6^. In a retrospective analysis, Pennington *et al*.^7^ have found that the ovarian cancer patients with germline or somatic mutations in 13 HR-associated genes (*BRCA1*, *BRCA2*, *ATM*, *BARD1*, *BRIP1*, *CHEK1*, *CHEK2*, *FAM175A*, *MRE11A*, *NBN*, *PALB2*, *RAD51C*, and *RAD51D*) had higher platinum sensitivity and prolonged overall survival than those without HR-associated gene mutations.

Clinical use of PARP inhibitors that induces synthetic lethality in HR deficient (HRD) cancer cells has a great impact on treatment strategies for ovarian cancer^8^. Niraparib maintenance therapy has shown prolonged progression-free survival (PFS) in platinum-sensitive, recurrent ovarian cancer patients with HRD^9^. Rucaparib maintenance has also improved PFS in platinum-sensitive, recurrent ovarian cancer patients with HRD^10^. However, the majority of subjects in clinical trials of PARP inhibitors were type II ovarian cancer patients^9–13^, and thus, the efficacy of PARP inhibitors for type I ovarian cancer, such as clear cell or low-grade endometrioid types, remains unclear. To date, the frequency of germline and somatic HR-associated gene mutations in type I ovarian cancer has yet to be elucidated fully.

In this study, we focused on difference in the distribution of ovarian cancer histologic subtypes between Western countries and Japan^14^. For example, clear cell carcinoma (CCC) accounts for 25% of ovarian cancer in Japan and less than 10% in the United States^15,16^. Therefore, we aimed to identify the frequency of germline and somatic HR-associated gene mutations per major histological subtypes of ovarian cancer in Japan, suggesting the therapeutic strategy of PARP inhibitors for ovarian cancers with HR-associated gene mutations.

## Results

### Clinicopathological characteristics of ovarian cancer patients

Clinicopathological characteristics of 207 patients (197 ovarian, 9 peritoneal, and 1 fallopian tube cancer) are shown in Table 1. Neoadjuvant chemotherapy cases that mainly consisted of HGSC patients were excluded. The median onset age of all patients was 56.0 years and more than half of patients (66%) were diagnosed at stage I. In all patients, the frequency of CCC (48%) was higher than that of HGSC (24%) that was the most common type of epithelial ovarian cancer^17^. Although more than half of HGSC and low-grade serous carcinoma (LGSC) patients were at stage III, over 60% of CCC, endometrioid carcinoma (EC), and mucinous carcinoma (MC) patients were at stage I.

**Table 1:** Clinical characteristics of different histological subtypes of ovarian cancer.

Histology	HGSC	CCC	EC	MC	LGSC
Number of patients	50	99	39	13	6
Median Age (range)	59.5 (38–84)	54 (35–82)	50 (35–78)	61 (32–89)	65.5 (32–75)
Stage	Number (%)				
I	7 (14.0)	61 (61.6)	28 (71.8)	10 (76.9)	2 (33.3)
II	9 (18.0)	12 (12.1)	5 (12.8)	0	0
III	29 (58.0)	20 (20.2)	4 (10.3)	3 (23.1)	4 (66.7)
IV	5 (10.0)	6 (6.1)	2 (5.1)	0	0
Somatic mutation					
*TP53*	41 (82.0)	5 (5.1)	9 (23.1)	8 (61.5)	3 (50.0)
*PIK3CA*	1 (2.0)	63 (63.6)	16 (41.0)	1 (7.7)	1 (16.7)
*ARID1A*	1 (2.0)	69 (69.7)	18 (46.2)	1 (7.7)	0
*KRAS*	1 (2.0)	14 (14.1)	18 (46.2)	8 (61.5)	0
*PTEN*	1 (2.0)	4 (4.0)	15 (38.5)	0	0

Next, we assessed mutation status of five genes (*TP53*, *ARID1A*, *PIK3CA*, *KRAS*, and *PTEN*), which were frequently mutated in ovarian cancer^18^, for each histologic type (Table 1). Most of HGSC (82%) and MC (62%) patients harbored *TP53* somatic mutations. CCC patients were characterized by high frequency of *ARID1A* (70%) and *PIK3CA* (64%) somatic mutations. EC patients harbored *KRAS* (46%) and *PTEN* (39%) somatic mutations in addition to *ARID1A* (46%) and *PIK3CA* (41%) somatic mutations.

### Landscape of HR-associated gene mutations in ovarian cancer

**Figure 1:**
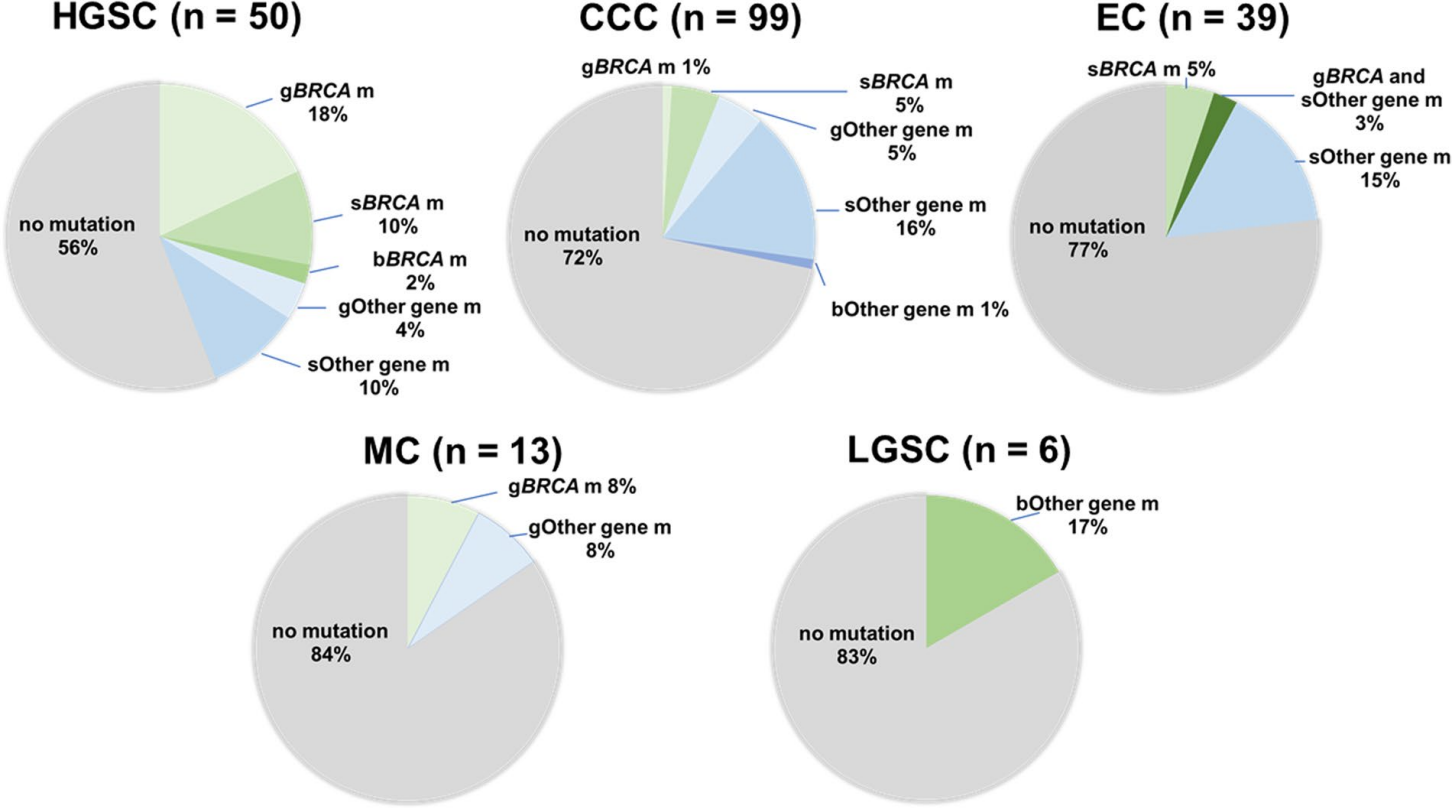
Frequency of HR-associated gene mutation based on histology. The frequency of HR-associated gene mutation based on histology is shown in each pie chart. The mutation data were classified into seven categories – germline *BRCA* mutation (g*BRCA* m), somatic *BRCA* mutation (s*BRCA* m), both germline and somatic *BRCA* mutation (b*BRCA* m), other germline gene mutation (gOther m), other somatic gene mutation (sOther m), other germline and somatic gene mutation (bOther m), and no mutation.

We investigated germline and somatic mutations of 16 HR-associated genes in 207 ovarian cancer samples. The average sequencing depth and the percentage of the target lesion that covered at least 20 reads were on average 98.6 and 98.9% in all samples, respectively. All the somatic mutations in HR-associated genes are listed in Supplementary Table S2. Missense mutation was the most frequent type of mutation (64%), followed by stopgain mutation (21%), frameshift insertion and deletion (11%), and splicing mutation (3%). Among 207 samples, 42 samples (20%) harbored at least one HR-associated gene mutation. The frequencies of germline and somatic HR-associated mutation in each histological subtype are shown in Fig. 1. Germline or somatic HR-associated gene mutations were detected in 44% of HGSC, 28% of CCC, 23% of EC, and 16% of MC, and 17% of LGSC patients. We investigated the correlation between stage and HR-associated gene mutations in each histological subtype (HGSC, CCC and EC). All ECs harboring HR-associated gene mutations or germline *BRCA* mutations were diagnosed as stage I. On the other hand, there was no obvious difference of HR-associated mutation frequency per stage in HGSC and CCC (Supplementary Fig. S1).

All germline mutations identified in our dataset are shown in Table 2. We detected 24 germline mutations in 22 ovarian cancer patients. Among the 24 germline HR-associated gene mutations, 12 (50%) were detected in HGSC patients, and almost all of them were either *BRCA1* or *BRCA2*. Intriguingly, *BRCA2* mutation was identified in one patient each of CCC, EC, and MC. When we focused on ovarian or breast cancer history in first- or second-degree relatives, 6 of 22 patients (27%) with a germline HR-associated gene mutation had family history with ovarian cancer or breast cancer. Of them, five patients were diagnosed with HGSC harboring germline *BRCA1/2* mutations.

**Table 2:** Germline variants and clinical features.

Patient ID	Age	Stage	Histological subtype	Gene	Class	Refseq ID	Nucleotide change	Amino acid change	Breast cancer patients in family	Ovarian cancer patients in family
13	46	I	HGSC	*BRCA1*	frameshift deletion	NM_007300	c.2028_2029del	p.T676fs	Yes	Yes
184	59	III	HGSC	*BRCA1*	stopgain	NM_007300	c.C2800T	p.Q934X	—	Yes
566	49	III	HGSC	*BRCA1*	splicing	NM_007300	c.212 + 2T > C		—	Yes
614	56	III	HGSC	*BRCA1*	frameshift deletion	NM_007300	c.321delT	p.F107fs	—	—
723	72	III	HGSC	*BRCA1*	stopgain	NM_007300	c.C2800T	p.Q934X	—	—
843	60	III	HGSC	*BRCA1*	splicing	NM_007300	c.5341–1G > A		—	Yes
584	75	III	HGSC	*BRCA2*	frameshift deletion	NM_000059	c.9127delG	p.E3043fs	—	—
1075	56	II	HGSC	*BRCA2*	frameshift insertion	NM_000059	c.845_846insTTTGG	p.H282fs	—	—
1300	57	IV	HGSC	*BRCA2*	stopgain	NM_000059	c.C9076T	p.Q3026X	—	—
1357	62	III	HGSC	*BRCA2*	stopgain	NM_000059	c.C6952T	p.R2318X	Yes	—
602	61	II	CCC	*BRCA2*	stopgain	NM_000059	c.C9076T	p.Q3026X	—	—
152	50	I	EC	*BRCA2*	frameshift deletion	NM_000059	c.5718_5719del	p.N1906fs	—	—
734	61	III	MC	*BRCA2*	frameshift deletion	NM_000059	c.958delC	p.L320fs	—	—
210	60	III	CCC	*ATM*	frameshift deletion	NM_000051	c.4799delT	p.V1600fs	—	—
292	52	I	CCC	*BRIP1*	splicing	NM_032043	c.2098–1G > A		—	—
396	32	I	MC	*CHEK1*	frameshift deletion	NM_001244846	c.668delA	p.E223fs	Yes	—
90	60	III	CCC	*EMSY*	Non-frameshift deletion	NM_001300943	c.3289_3291del	p.1097_1097del	—	—
1133	59	II	HGSC	*RAD51D*	frameshift insertion	NM_002878	c.271_272insT	p.K91fs	—	—
237	86	III	CCC	*RAD51D*	frameshift insertion	NM_002878	c.271_272insTA	p.K91fs	—	—
390	68	I	CCC	*RAD51D*	frameshift insertion	NM_002878	c.271_272insTA	p.K91fs	—	—
184	59	III	HGSC	*RAD54L*	frameshift deletion	NM_003579	c.1961delG	p.R654fs	—	—
90	60	III	CCC	*RAD54L*	stopgain	NM_003579	c.1092_1093insC	p.G364_R	—	—
261	53	III	CCC	*RAD54L*	stopgain	NM_003579	c.1092_1093insC	p.G364_R	—	—
994	68	I	LGSC	*RAD54L*	stopgain	NM_003579	c.1092_1093insC	p.G364_R	—	—

### Somatic HR-associated gene mutations per histological subtype

**Figure 2:**
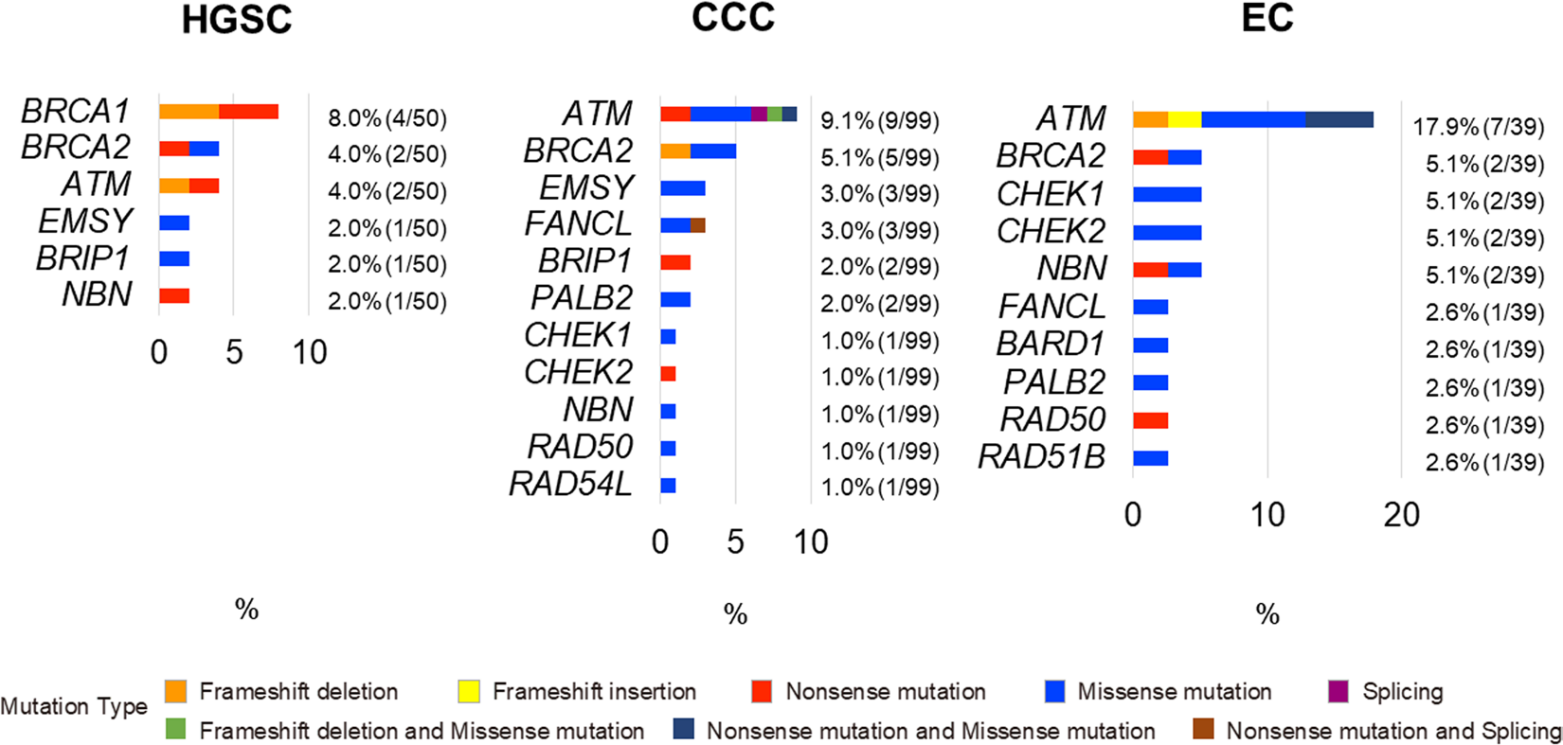
Details of somatic HR-associated gene mutations in HGSC, CCC, and EC. The frequency of HR-associated gene mutations per each histological subtype is shown. The details of mutation types are also shown.

Next, we compared the frequency of HR-associated gene alterations among three major histological subtypes (HGSC, CCC, and EC) of ovarian cancer (Fig. 2). *BRCA1/2* somatic mutations were detected more frequently in HGSC patients (12%) than in CCC (5%) or EC patients (5%). However, *ATM* somatic mutations were detected more frequently in CCC (9%) and EC patients (18%) than in HGSC patients (4%). Most of the other HR-associated gene mutations were detected in a small population of each histological subtype.

### Clinical significance of HR-associated gene mutations

**Figure 3:**
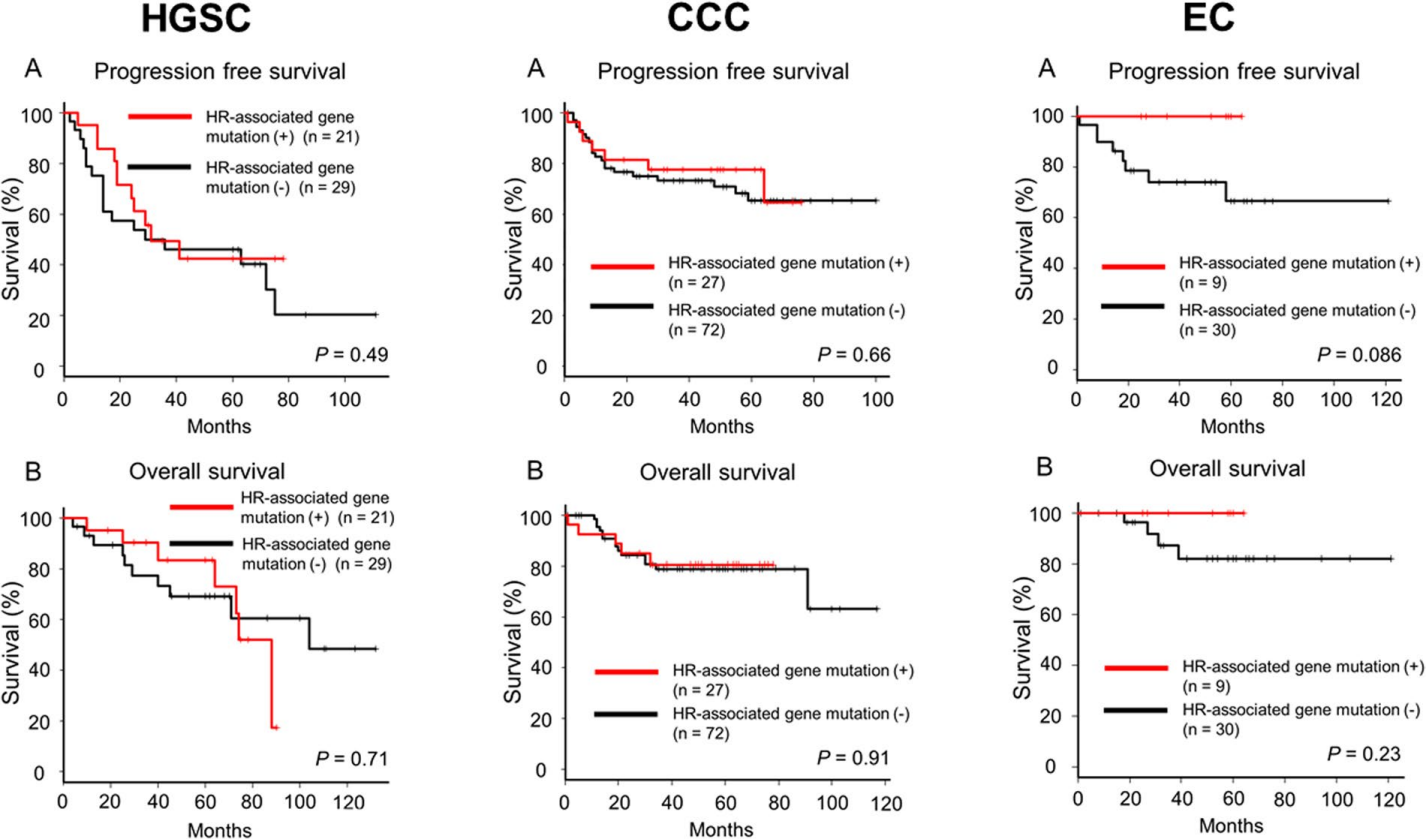
Association between HR-associated gene alterations and clinical outcome in HGSC, CCC, and EC. Kaplan–Meier estimates of progression-free survival (**A**) and overall survival (**B**) in HGSC, CC, and EC.

We divided HGSC, CCC, and EC into two subgroups based on the status of HR-associated gene mutation and compared clinical characteristics between two subgroups in each histological subtype (Supplementary Table S3). EC patients with HR-associated gene mutation had younger age of onset than those without HR-associated gene mutations. No significant prognostic difference was observed between patients with and without HR-associated gene mutation irrespective of histology (Fig. 3). When we focused on only *BRCA1/2* mutations, there were no significant differences in progression-free or overall survival between patients with and without *BRCA1/2* mutations (Supplementary Fig. S2).

### Correlation between mismatch repair gene mutations and HR-associated gene mutations

We investigated germline and somatic mutations of four mismatch repair (MMR) genes (*MLH1*, *MSH2*, *MSH6*, and *PMS2*) in 207 ovarian cancer cases (Table 3). Ten patients (5%) harbored deleterious germline or somatic mutations in MMR genes. The frequency of MMR gene mutation was 2% (HGSC), 3% (CCC), and 10% (EC) and there was no significant difference in the frequency of MMR gene mutation among histological subtypes. Subsequently, we investigated the correlation between MMR and HR-associated gene mutations. More than half of HR-associated gene mutation and MMR gene mutation were somatic mutations (63.3% and 70%) (Supplementary Fig. S3A). Patients with germline or somatic MMR gene mutation exhibited significantly higher frequency of germline or somatic HR-associated gene mutations (p = 0.0072) (Supplementary Fig. S3B).

**Table 3:** Germline and somatic mutations of MMR genes.

	Patient ID	Age	Stage	Histological subtype	Gene	Class	Refseq ID	Nucleotide change	Amino acid change	HR-associated gene mutation
Germline	762	38	I	EC	*MSH2*	frameshift insertion	NM_000251	c.131_132insG	p.T44fs	sATM, sPALB2
	509	47	I	EC	*MSH6*	stopgain	NM_001281492	c.C583T	p.Q195X	sATM, sCHEK1
	329	59	II	HGSC	*MSH6*	frameshift insertion	NM_001281492	c.3692_3693insG	p.X1231delinsX	sBRCA1
Somatic	378	53	II	CCC	*MLH1*	missense mutation	NM_000249	c.C350T	p.T117M	—
	519	37	III	CCC	*MLH1*	missense mutation	NM_001167617	c.C688G	p.Q230E	sATM
	555	50	III	HGSC	*MLH1*	missense mutation	NM_000249	c.T107C	p.I36T	—
	646	46	I	EC	*MLH1*	missense mutation	NM_000249	c.A525C	p.K175N	sBRCA2, sATM, sBARD1, sCHEK1, sCHEK2, sNBN, sRAD50
	762	38	I	EC	*MSH2*	frameshift deletion	NM_000251	c.1706delA	p.E569fs	sATM, sPALB2
	1031	43	I	CCC	*MSH2*	frameshift deletion	NM_001258281	c.1403delG	p.R468fs	—
	1146	50	I	EC	*MSH2*	frameshift deletion	NM_000251	c.67delT	p.F23fs	sATM, sFANCL
	762	38	I	EC	*MSH6*	frameshift deletion	NM_000179	c.3254delC	p.T1085fs	sATM, sPALB2
	509	47	I	EC	*MSH6*	frameshift deletion	NM_001281492	c.297delG	p.E99fs	sATM, sCHEK1
	762	38	I	EC	*PMS2*	stopgain	NM_000535	c.C1882T	p.R628X	sATM, sPALB2
	1321	41	I	EC	*PMS2*	missense mutation	NM_000535	c.C637T	p.P213S	sBRCA2, sCHEK2, sRAD51B

## Discussion

In this study, we demonstrated the frequency of germline and somatic HR-associated gene mutations in Japanese patients with ovarian cancer. In Japan, three studies^19–21^ have clarified the frequency of germline *BRCA1/2* mutation in ovarian cancer but not somatic *BRCA1/2* mutation. Pennington *et al*.^7^ have assessed the frequency of germline and somatic HR-associated gene mutations in 390 ovarian cancer dataset largely composed of HGSC. The Cancer Genome Atlas (TCGA) network^4^ has performed exome sequencing and clarified the frequency of germline and somatic HR-associated gene mutations in 316 HGSC cases. These studies have demonstrated that HR-associated gene mutations might correlate with better prognosis of HGSC patients. On the other hand, there was no report to assess the frequency and clinical significance of germline and somatic HR-associated gene mutations in a large scale of non-HGSC cases. Therefore, we focused on difference in the frequency of germline and somatic HR-associated gene mutations among major histological types of ovarian cancer.

Major histological subtypes of ovarian cancer are characterized by several cancer-associated gene mutations^22^. For example, *TP53* mutation was detected in 97% of HGSC patients^4^ and more than 50% of MC patients^23,24^. *KRAS* mutation was found in 50% of MC and 18% of LGSC^25^ but not in HSGC. Similarly, *ARID1A* and *PIK3CA* were frequently mutated in CCC and EC patients but not in HGSC^26–28^. Our result was almost consistent with those of previous studies. Although the distribution of ovarian cancer histological subtypes is different between Japan and Western countries, representative mutation profiles per histological subtype are similar beyond ethnicity.

Corresponding to previous studies^4,7^, our findings showed that germline and somatic *BRCA1/2* mutations were frequently identified in HGSC patients. Somatic *BRCA1/2* mutations were also identified in a part of CCC (5%) and EC (5%). Interestingly, germline *BRCA2* mutation was identified in one sample each of CCC, EC, and MC. HR-associated gene mutations were also detected in HGSC, CCC, and EC samples^24,29^. Surprisingly, our data indicated that a small part of the MC patients harbored HR-associated gene mutations and thereby, PARP inhibitors might be a potent therapeutic alternative for these cancers. However, the sample size of MC patients in this study was small (n = 13). Further analysis will be needed to elucidate the clinical significance of HR-associated gene mutations in MC, which is usually refractory to conventional platinum-taxane chemotherapy^30^.

The frequency of somatic *ATM* mutation was higher in CCC and EC than in other histological subtypes. The ATM protein kinase plays an important role in DNA damage response^31^. Genetic alterations of *ATM* were found in many cancers^32^, such as colorectal cancer (10%), prostate cancer (8.8%), lung cancer (7.3%), and ovarian cancer (4.5%). The frequency of *ATM* mutation in CCC was previously reported to be 7% (3/48)^27^. *ATM*-deficient tumor cells, such as mantle cell lymphoma^33^ and colorectal cancer^32^ exhibited high sensitivity to the PARP inhibitor, olaparib *in vitro* and in *vivo*^34^. In a phase 2 clinical trial to assess the effectiveness of olaparib for metastatic prostate cancer and gastric cancer, *ATM* mutated cases had better prognosis than *ATM* wildtype cases^35,36^. Therefore, PARP inhibitors might provide a survival advantage to *ATM*-mutated ovarian cancer, especially for CCC and EC types and tumor sequencing might be important not to miss these somatic mutations,.

Mismatch repair (MMR) is a repair system of base mismatch pairing caused in DNA replication, and MMR genes (*MLH1*, *MSH2*, *MSH6*, and *PMS2*) often react to errors of a DNA single strand break. MMR gene mutations lead to microsatellite instability (MSI), and loss of function in germline MMR gene alterations cause Lynch syndrome^37^. PD-1 antibody contributes to a favorable benefit for patients with MSI-high cancer^38^. Our results showed positive correlation between mutation status of HR-associated genes and mismatch repair genes. It is inconclusive that defective MMR might contribute to platinum sensitivity for HGSC^39^. However, Fleury *et al*. have reported that downregulation of both HR pathway and MMR pathway increases sensitivity of PARP inhibitor^40^. Furthermore, the phase 2 study for advanced or metastatic triple-negative breast cancer has revealed that combination therapy of Niraparib with Pembrolizumab confers higher response rates in patients with tumor *BRCA* mutation^41^. Therefore, clinical trials evaluating the efficacy of PARP inhibitor and PD-1 antibody combination should be conducted for ovarian cancer patients.

Our findings indicated the possibility of HRD in major histological types of ovarian cancer. When we divided each histological group into two subgroups on the basis of HR-associated gene status in this study, there were no significant difference in clinical outcome between two subgroups. This might be due to several reasons. First, most of patients in this study were stage 1. Second, each HR-associated gene mutation was not evaluated at protein level for its pathogenicity. At least, we might need to evaluate an association of HR-associated gene mutation with other HRD assessment such as HRD score^42^ and HRDetect^43^. Third, the sample size of each histological subtype was still small, and we could not exclude any influence of other prognostic factors such as stage, residual disease, and chemotherapy.

In conclusion, we elucidated the mutation profile of HR-associated genes in major histological types of ovarian cancer. PARP inhibitors might provide survival advantage to ovarian cancer patients with HR-associated gene mutations beyond histological subtypes.

## Methods

### Clinical specimens

This study was performed in conformity with the Declaration of Helsinki and approved by the institutional ethics review board at Niigata University (G2016-0006). The subjects were 207 epithelial ovarian cancer patients who had undergone initial surgery between July 2006 and September 2017 at Niigata University Medical and Dental Hospital^44^. During the study period, we had enrolled 376 epithelial ovarian cancer patients. At first, 41 cases receiving neoadjuvant chemotherapy were excluded from this study. Then, 128 cases that could not provide the required DNA quality for blood and/or tumor samples were excluded. Finally, 207 epithelial ovarian cancer samples were collected as a cohort. All patients provided written informed consent for sample collection and subsequent analysis. Fresh-frozen tissue samples were obtained from primary tumor tissues. Staging of ovarian cancer was done following the criteria of the International Federation of Gynecology and Obstetrics (FIGO)^45^. Histological diagnosis was performed by two gynecological pathologists belonging to the Japanese Society of Pathology and assessed on formalin-fixed and paraffin-embedded hematoxylin and eosin sections. Histological subtypes were diagnosed according to WHO classification of ovarian tumors^46^. Tumor DNA was extracted from frozen tissues containing more than 70% of tumor cells, revealed by histological evaluation. PFS time was evaluated for the interval from primary surgery of a disease to disease progression or recurrence. Disease progression was defined as at least a 20% growth in the size of the tumor or the longest diameters of lesions or as the appearance of one or more new lesions and/or unequivocal progression existing non-target lesions since primary surgery following standard Response Evaluation Criteria In Solid Tumors (RECIST) guidelines^47^. Overall survival time was evaluated for the interval from primary surgery to the death by ovarian cancer.

### DNA extraction

Tumor DNA extraction was performed with Tissue Genomic DNA Extraction Mini Kit (FAVORGEN), according to the manufacturer’s instructions. Blood DNA was extracted with the QIAamp DNA Blood Maxi kit (Qiagen) following the manufacturer’s instructions. Genomic DNA was quantified using a Qubit dsDNA HS Assay Kit (Thermo Fisher Scientific).

### Targeted sequencing

Targeted sequencing of 16 HR-associated genes, 5 ovarian cancer-associated genes, and 4 mismatch repair genes (Supplementary Table S1) was conducted with the pre-capture pooling method described in our previous study^48^. In summary, 20 ng of DNA was simultaneously fragmented and adapter-ligated with a SureSelect QXT Library Prep Kit (Agilent Technologies). The fragmented libraries with distinct indexed adapters were preserved at equimolar amounts. Subsequently, target enrichment was performed using the SeqCap EZ Choice System (Roche Diagnostics). A DNA probe set complementary to the target region was selected by NimbleDesign (https://design.nimblegen.com). The libraries were sequenced on a MiSeq platform (Illumina). The paired-end read data were aligned to a human reference genome (hg19) using BWA^49^ and SAMtools^50^. The aligned reads were processed for removal of PCR duplicates using Picard tools (v.1.111; broadinstitute.github.io/picard). Local realignments and base-quality recalibrations were conducted using GATK (v.3.2.2)^51,52^. The averages of depth and the coverages were calculated by the DepthOfCoverage and CallableLoci tools in GATK. Somatic single nucleotide variants (SNVs) and short insertions and deletions (indels) were called in each pair of tumor and matched normal blood samples using Strelka (v.1.0.14) workflow software^53^. Sites with a depth greater than or equal to 20 in both tumor and matching normal blood samples, were subjected to somatic variant calling. We set the quality-score threshold to greater than or equal to 30 for SNVs and 50 for indels. Functional annotation of the identified somatic variants was implemented by ANNOVAR^54^. The prevalence of somatic mutations indicated in previous genome-wide screenings in various cancer types were collected from the COSMIC database (v.79)^55^. The detected variants in germline *BRCA1/2* were interpreted using BRCA Exchange^56^ and confirmed that there was no pathogenic germline missense *BRCA1/2* mutation. In germline mutation analysis, stopgain, frameshift, and splicing mutations were used. In addition to these mutation types, non-frameshift indel and missense mutations were used in somatic mutation analysis.

### Statistical analysis

All computations were conducted using R. Standard statistical tests were used as appropriate, including unpaired t-test, Welch’s exact test, and logrank test.

## Supplementary information

Supplementary information

## Data Availability

The datasets generated during and/or analyzed during the current study are available from the corresponding author on reasonable request.

## References

[1] Alexandrov LB (2013). Signatures of mutational processes in human cancer. Nature.

[2] Knijnenburg TA (2018). Genomic and Molecular Landscape of DNA Damage Repair Deficiency across The Cancer Genome Atlas. Cell Rep.

[3] Konstantinopoulos PA, Ceccaldi R, Shapiro GI, D’Andrea AD (2015). Homologous Recombination Deficiency: Exploiting the Fundamental Vulnerability of Ovarian Cancer. Cancer Discov.

[4] Cancer Genome Atlas Research N (2011). Integrated genomic analyses of ovarian carcinoma. Nature.

[5] Tumiati M (2018). A Functional Homologous Recombination Assay Predicts Primary Chemotherapy Response and Long-Term Survival in Ovarian Cancer Patients. Clin. Cancer Res..

[6] Bolton KL (2012). Association between BRCA1 and BRCA2 mutations and survival in women with invasive epithelial ovarian cancer. JAMA.

[7] Pennington KP (2014). Germline and somatic mutations in homologous recombination genes predict platinum response and survival in ovarian, fallopian tube, and peritoneal carcinomas. Clin Cancer Res.

[8] Lord CJ, Ashworth A (2017). PARP inhibitors: Synthetic lethality in the clinic. Science.

[9] Mirza MR (2016). Niraparib Maintenance Therapy in Platinum-Sensitive, Recurrent Ovarian Cancer. N Engl J Med.

[10] Coleman RL (2017). Rucaparib maintenance treatment for recurrent ovarian carcinoma after response to platinum therapy (ARIEL3): a randomised, double-blind, placebo-controlled, phase 3 trial. Lancet.

[11] Kurman RJ, Shih IM (2010). The origin and pathogenesis of epithelial ovarian cancer: a proposed unifying theory. Am. J. Surg. Pathol..

[12] Pujade-Lauraine E (2017). Olaparib tablets as maintenance therapy in patients with platinum-sensitive, relapsed ovarian cancer and a BRCA1/2 mutation (SOLO2/ENGOT-Ov21): a double-blind, randomised, placebo-controlled, phase 3 trial. Lancet Oncol.

[13] Moore K (2018). Maintenance Olaparib in Patients with Newly Diagnosed Advanced Ovarian Cancer. N Engl J Med.

[14] Matz M (2017). Worldwide comparison of ovarian cancer survival: Histological group and stage at diagnosis (CONCORD-2). Gynecol Oncol.

[15] Sugiyama T (2000). Clinical characteristics of clear cell carcinoma of the ovary: a distinct histologic type with poor prognosis and resistance to platinum-based chemotherapy. Cancer.

[16] Konstantinopoulos PA (2018). Phase II study of single-agent cabozantinib in patients with recurrent clear cell ovarian, primary peritoneal or fallopian tube cancer (NRG-GY001). Gynecol Oncol.

[17] Torre LA (2018). Ovarian cancer statistics, 2018. CA Cancer J Clin.

[18] Rojas Veronica, Hirshfield Kim, Ganesan Shridar, Rodriguez-Rodriguez Lorna (2016). Molecular Characterization of Epithelial Ovarian Cancer: Implications for Diagnosis and Treatment. International Journal of Molecular Sciences.

[19] Sakamoto I (2016). BRCA1 and BRCA2 mutations in Japanese patients with ovarian, fallopian tube, and primary peritoneal cancer. Cancer.

[20] Hirasawa A (2017). Prevalence of pathogenic germline variants detected by multigene sequencing in unselected Japanese patients with ovarian cancer. Oncotarget.

[21] Enomoto T (2019). The first Japanese nationwide multicenter study of BRCA mutation testing in ovarian cancer: CHARacterizing the cross-sectionaL approach to Ovarian cancer geneTic TEsting of BRCA (CHARLOTTE). Int J Gynecol Cancer.

[22] Ramirez O, Vaughan C, Herrera G, Guries R (2011). Temporal and spatial resource use by female three-toed sloths and their young in an agricultural landscape in Costa Rica. Rev Biol Trop.

[23] Ryland GL (2015). Mutational landscape of mucinous ovarian carcinoma and its neoplastic precursors. Genome Med.

[24] Mueller JJ (2018). Massively parallel sequencing analysis of mucinous ovarian carcinomas: genomic profiling and differential diagnoses. Gynecol Oncol.

[25] Jones S (2012). Low-grade serous carcinomas of the ovary contain very few point mutations. J Pathol.

[26] Itamochi H (2017). Whole-genome sequencing revealed novel prognostic biomarkers and promising targets for therapy of ovarian clear cell carcinoma. Br. J. Cancer.

[27] Shibuya Y (2018). Identification of somatic genetic alterations in ovarian clear cell carcinoma with next generation sequencing. Genes Chromosomes Cancer.

[28] Wiegand KC (2010). ARID1A mutations in endometriosis-associated ovarian carcinomas. N Engl J Med.

[29] Hanley GE (2018). A population-based analysis of germline BRCA1 and BRCA2 testing among ovarian cancer patients in an era of histotype-specific approaches to ovarian cancer prevention. BMC Cancer.

[30] Ricci Francesca, Affatato Roberta, Carrassa Laura, Damia Giovanna (2018). Recent Insights into Mucinous Ovarian Carcinoma. International Journal of Molecular Sciences.

[31] Lieber MR (2010). The mechanism of double-strand DNA break repair by the nonhomologous DNA end-joining pathway. Annu Rev Biochem.

[32] Wang C, Jette N, Moussienko D, Bebb DG, Lees-Miller SP (2017). ATM-Deficient Colorectal Cancer Cells Are Sensitive to the PARP Inhibitor Olaparib. Transl Oncol.

[33] Weston VJ (2010). The PARP inhibitor olaparib induces significant killing of ATM-deficient lymphoid tumor cells *in vitro* and *in vivo*. Blood.

[34] Murai J (2012). Trapping of PARP1 and PARP2 by Clinical PARP Inhibitors. Cancer Res.

[35] Mateo J (2015). DNA-Repair Defects and Olaparib in Metastatic Prostate Cancer. N Engl J Med.

[36] Bang YJ (2015). Randomized, Double-Blind Phase II Trial With Prospective Classification by ATM Protein Level to Evaluate the Efficacy and Tolerability of Olaparib Plus Paclitaxel in Patients With Recurrent or Metastatic Gastric Cancer. J Clin Oncol.

[37] Zhao H (2014). Mismatch repair deficiency endows tumors with a unique mutation signature and sensitivity to DNA double-strand breaks. Elife.

[38] Yi M (2018). Biomarkers for predicting efficacy of PD-1/PD-L1 inhibitors. Mol Cancer.

[39] Helleman J (2006). Mismatch repair and treatment resistance in ovarian cancer. BMC Cancer.

[40] Fleury H (2017). Cumulative defects in DNA repair pathways drive the PARP inhibitor response in high-grade serous epithelial ovarian cancer cell lines. Oncotarget.

[41] Vinayak Shaveta, Tolaney Sara M., Schwartzberg Lee, Mita Monica, McCann Georgia, Tan Antoinette R., Wahner-Hendrickson Andrea E., Forero Andres, Anders Carey, Wulf Gerburg M., Dillon Patrick, Lynce Filipa, Zarwan Corrine, Erban John K., Zhou Yinghui, Buerstatte Nathan, Graham Julie R., Arora Sujata, Dezube Bruce J., Telli Melinda L. (2019). Open-label Clinical Trial of Niraparib Combined With Pembrolizumab for Treatment of Advanced or Metastatic Triple-Negative Breast Cancer. JAMA Oncology.

[42] Hodgson DR (2018). Candidate biomarkers of PARP inhibitor sensitivity in ovarian cancer beyond the BRCA genes. Br J Cancer.

[43] Davies H (2017). HRDetect is a predictor of BRCA1 and BRCA2 deficiency based on mutational signatures. Nat Med.

[44] Yoshihara K (2012). High-risk ovarian cancer based on 126-gene expression signature is uniquely characterized by downregulation of antigen presentation pathway. Clin Cancer Res.

[45] Mutch DG, Prat J (2014). 2014 FIGO staging for ovarian, fallopian tube and peritoneal cancer. Gynecol Oncol.

[46] Meinhold-Heerlein I (2016). Erratum to: The new WHO classification of ovarian, fallopian tube, and primary peritoneal cancer and its clinical implications. Arch Gynecol Obstet.

[47] Eisenhauer EA (2009). New response evaluation criteria in solid tumours: revised RECIST guideline (version 1.1). Eur J Cancer.

[48] Ahmadloo S (2017). Rapid and cost-effective high-throughput sequencing for identification of germline mutations of BRCA1 and BRCA2. J Hum Genet.

[49] Li H, Durbin R (2009). Fast and accurate short read alignment with Burrows-Wheeler transform. Bioinformatics.

[50] Li H (2009). The Sequence Alignment/Map format and SAMtools. Bioinformatics.

[51] DePristo MA (2011). A framework for variation discovery and genotyping using next-generation DNA sequencing data. Nat Genet.

[52] McKenna A (2010). The Genome Analysis Toolkit: a MapReduce framework for analyzing next-generation DNA sequencing data. Genome Res.

[53] Saunders CT (2012). Strelka: accurate somatic small-variant calling from sequenced tumor-normal sample pairs. Bioinformatics.

[54] Wang K, Li M, Hakonarson H (2010). ANNOVAR: functional annotation of genetic variants from high-throughput sequencing data. Nucleic Acids Res.

[55] Forbes SA (2015). COSMIC: exploring the world’s knowledge of somatic mutations in human cancer. Nucleic Acids Res.

[56] Cline, M. S. *et al*. BRCA Challenge: BRCA Exchange as a global resource for variants in BRCA1 and BRCA2. *PLoS Genet* **14**, e1007752, doi:10.1371/journal.pgen.1007752 (2018). 10.1371/journal.pgen.1007752 PMC6324924 30586411

