# Supplementary material for: Germline and somatic mutations of homologous recombination-associated genes in Japanese ovarian cancer patients
Source: Sci Rep. 2019 Nov 28;9:17808. doi: 10.1038/s41598-019-54116-y (PMC6882827; doi:10.1038/s41598-019-54116-y)
Supplement: Supplementary file 1 — Supplementary information [file 41598_2019_54116_MOESM1_ESM.docx]

**Germline and somatic mutations in homologous recombination associated genes in Japanese ovarian cancer patients**

Kentaro Sugino^1*^, Ryo Tamura^1*^, Hirofumi Nakaoka^2*^, Nozomi Yachida^1^, Manako Yamaguchi^1^, Yutaro Mori^1^, Kaoru Yamawaki^1^, Kazuaki Suda^1^, Tatsuya Ishiguro^1^, Sosuke Adachi^1^, Masanori Isobe^1^, Masayuki Yamaguchi^1^, Katsunori Kashima^1^, Teiichi Motoyama^3^, Ituro Inoue^2^, Kosuke Yoshihara^1†^, Takayuki Enomoto^1^

1. Department of Obstetrics and Gynecology, Niigata University Graduate School of Medical and Dental Sciences, Niigata 951-8510, Japan.
2. Human Genetics Laboratory, National Institute of Genetics, Mishima 411-8540, Japan.
3. Department of Molecular and Diagnostic Pathology, Niigata University Graduate School of Medical and Dental Sciences, Niigata 951-8510, Japan

**Supplementary Table S1. A list of targeted genes**

| Gene Symbol | Gene Name | Gene ID | Gene Title |
| --- | --- | --- | --- |
| *ATM* | ATM serine/threonine kinase | 472 | HR-associated gene |
| *BARD1* | BRCA1 associated RING domain 1 | 580 | HR-associated gene |
| *BRCA1* | BRCA1, DNA repair associated | 672 | HR-associated gene |
| *BRCA2* | BRCA2, DNA repair associated | 675 | HR-associated gene |
| *BRIP1* | BRCA1 interacting protein C-terminal helicase 1 | 83990 | HR-associated gene |
| *CHEK1* | checkpoint kinase 1 | 1111 | HR-associated gene |
| *CHEK2* | checkpoint kinase 2 | 11200 | HR-associated gene |
| *EMSY* | EMSY, BRCA2 interacting transcriptional repressor | 56946 | HR-associated gene |
| *FANCL* | Fanconi anemia complementation group L | 55120 | HR-associated gene |
| *NBN* | nibrin | 4683 | HR-associated gene |
| *PALB2* | partner and localizer of BRCA2 | 79728 | HR-associated gene |
| *RAD50* | RAD50 double strand break repair protein | 10111 | HR-associated gene |
| *RAD51B* | RAD51 paralog B | 5890 | HR-associated gene |

**Supplementary Table S1. Continued.**

| Gene Symbol | Gene Name | Gene ID | Gene Title |
| --- | --- | --- | --- |
| *RAD51C* | RAD51 paralog C | 5889 | HR-associated gene |
| *RAD51D* | RAD51 paralog D | 5892 | HR-associated gene |
| *RAD54L* | RAD54-like (S. cerevisiae) | 8438 | HR-associated gene |
| *TP53* | tumor protein p53 | 7157 | Ovarian cancer biomarker |
| *PTEN* | phosphatase and tensin homolog | 5728 | Ovarian cancer biomarker |
| *ARID1A* | AT-rich interaction domain 1A | 8289 | Ovarian cancer biomarker |
| *PIK3CA* | phosphatidylinositol-4,5-bisphosphate 3-kinase catalytic subunit alpha | 5290 | Ovarian cancer biomarker |
| *KRAS* | KRAS proto-oncogene, GTPase | 3845 | Ovarian cancer biomarker |
| *MLH1* | mutL homolog 1 | 4292 | Mismatch repair gene |
| *MSH2* | mutS homolog 2 | 4436 | Mismatch repair gene |
| *MSH6* | mutS homolog 6 | 2956 | Mismatch repair gene |
| *PMS2* | PMS1 homolog 2, mismatch repair system component | 5395 | Mismatch repair gene |

**Supplementary Table S2. A list of somatic variants**

| Patient ID | Age | Stage | Histological subtype | Gene | Class | Refseq ID | Nucleotide change | Amino acid change |
| --- | --- | --- | --- | --- | --- | --- | --- | --- |
| 180 | 81 | Ⅲ | HGSC | *BRCA1* | stopgain | NM_007298 | c.C1018T | p.R340X |
| 209 | 58 | Ⅲ | HGSC | *BRCA1* | frameshift deletion | NM_007300 | c.3818_3840del | p.Q1273fs |
| 329 | 59 | Ⅱ | HGSC | *BRCA1* | stopgain | NM_007294 | c.C3607T | p.R1203X |
| 1063 | 76 | Ⅱ | HGSC | *BRCA1* | frameshift deletion | NM_007300 | c.3840_3841del | p.S1280fs |
| 635 | 48 | Ⅰ | CCC | *BRCA2* | missense mutation | NM_000059 | c.T4284A | p.F1428L |
| 646 | 46 | Ⅰ | EC | *BRCA2* | stopgain | NM_000059 | c.G9946T | p.E3316X |
| 993 | 59 | Ⅰ | CCC | *BRCA2* | frameshift deletion | NM_000059 | c.965_968del | p.K322fs |
| 1027 | 37 | Ⅱ | CCC | *BRCA2* | missense mutation | NM_000059 | c.C3422T | p.T1141I |
| 1027 | 37 | Ⅱ | CCC | *BRCA2* | missense mutation | NM_000059 | c.T3641C | p.V1214A |
| 1027 | 37 | Ⅱ | CCC | *BRCA2* | missense mutation | NM_000059 | c.C9604T | p.P3202S |
| 1075 | 56 | Ⅱ | HGSC | *BRCA2* | missense mutation | NM_000059 | c.A2944T | p.I982L |
| 1265 | 45 | Ⅰ | CCC | *BRCA2* | frameshift deletion | NM_000059 | c.2665delA | p.N889fs |
| 1266 | 72 | Ⅲ | CCC | *BRCA2* | missense mutation | NM_000059 | c.C7127T | p.A2376V |
| 1290 | 58 | Ⅰ | HGSC | *BRCA2* | stopgain | NM_000059 | c.G6385T | p.E2129X |
| 1321 | 41 | Ⅰ | EC | *BRCA2* | missense mutation | NM_000059 | c.G172A | p.E58K |
| 1321 | 41 | Ⅰ | EC | *BRCA2* | missense mutation | NM_000059 | c.T7156G | p.S2386A |
| 148 | 45 | Ⅰ | EC | *ATM* | missense mutation | NM_000051 | c.G5044C | p.D1682H |
| 152 | 50 | Ⅰ | EC | *ATM* | missense mutation | NM_000051 | c.G4999A | p.V1667I |

**Supplementary Table S2. Continued.**

| Patient ID | Age | Stage | Histological subtype | Gene | Class | Refseq ID | Nucleotide change | Amino acid change |
| --- | --- | --- | --- | --- | --- | --- | --- | --- |
| 173 | 45 | Ⅰ | EC | *ATM* | missense mutation | NM_000051 | c.C2804T | p.T935M |
| 173 | 45 | Ⅰ | EC | *ATM* | stopgain | NM_000051 | c.C6889T | p.Q2297X |
| 204 | 47 | Ⅰ | CCC | *ATM* | missense mutation | NM_000051 | c.T6488G | p.L2163R |
| 215 | 56 | Ⅲ | HGSC | *ATM* | stopgain | NM_000051 | c.C1396T | p.Q466X |
| 237 | 86 | Ⅲ | CCC | *ATM* | missense mutation | NM_000051 | c.T3454C | p.S1152P |
| 509 | 47 | Ⅰ | EC | *ATM* | stopgain | NM_000051 | c.C67T | p.R23X |
| 509 | 47 | Ⅰ | EC | *ATM* | missense mutation | NM_000051 | c.C9022T | p.R3008C |
| 519 | 37 | Ⅲ | CCC | *ATM* | stopgain | NM_000051 | c.C7327T | p.R2443X |
| 646 | 46 | Ⅰ | EC | *ATM* | missense mutation | NM_000051 | c.C3790A | p.H1264N |
| 673 | 48 | Ⅱ | CCC | *ATM* | stopgain | NM_000051 | c.C5623T | p.R1875X |
| 762 | 38 | Ⅰ | EC | *ATM* | frameshift insertion | NM_000051 | c.4736dupA | p.Q1579fs |
| 902 | 56 | Ⅰ | CCC | *ATM* | missense mutation | NM_000051 | c.A348C | p.K116N |
| 902 | 56 | Ⅰ | CCC | *ATM* | frameshift deletion | NM_000051 | c.345delA | p.L115fs |
| 949 | 45 | Ⅰ | CCC | *ATM* | missense mutation | NM_000051 | c.C2280G | p.I760M |
| 949 | 45 | Ⅰ | CCC | *ATM* | stopgain | NM_000051 | c.C2426G | p.S809X |
| 994 | 68 | Ⅰ | LGSC | *ATM* | missense mutation | NM_000051 | c.A7343G | p.D2448G |
| 995 | 41 | Ⅰ | CCC | *ATM* | missense mutation | NM_000051 | c.A967G | p.I323V |
| 1096 | 49 | Ⅰ | HGSC | *ATM* | frameshift deletion | NM_000051 | c.5189delG | p.R1730fs |
| 1146 | 50 | Ⅰ | EC | *ATM* | frameshift deletion | NM_000051 | c.4709delT | p.V1570fs |

**Supplementary Table S2. Continued.**

| Patient ID | Age | Stage | Histological subtype | Gene | Class | Refseq ID | Nucleotide change | Amino acid change |
| --- | --- | --- | --- | --- | --- | --- | --- | --- |
| 1336 | 57 | Ⅰ | CCC | *ATM* | missense mutation | NM_000051 | c.G6490A | p.E2164K |
| 1333 | 72 | Ⅲ | CCC | *ATM* | splicing | NM_000051 | c.5005+1G>T |  |
| 646 | 46 | Ⅰ | EC | *BARD1* | missense mutation | NM_001282543 | c.A1377C | p.E459D |
| 840 | 60 | Ⅱ | HGSC | *BRIP1* | missense mutation | NM_032043 | c.G2278A | p.V760I |
| 1027 | 37 | Ⅱ | CCC | *BRIP1* | missense mutation | NM_032043 | c.A1077G | p.I359M |
| 1030 | 47 | Ⅰ | CCC | *BRIP1* | missense mutation | NM_032043 | c.C856T | p.P286S |
| 509 | 47 | Ⅰ | EC | *CHEK1* | missense mutation | NM_001274 | c.G1175A | p.C392Y |
| 646 | 46 | Ⅰ | EC | *CHEK1* | missense mutation | NM_001114121 | c.A586C | p.I196L |
| 1027 | 37 | Ⅱ | CCC | *CHEK1* | missense mutation | NM_001114121 | c.A991G | p.S331G |
| 646 | 46 | Ⅰ | EC | *CHEK2* | missense mutation | NM_007194 | c.G542A | p.R181H |
| 1263 | 48 | Ⅰ | CCC | *CHEK2* | stopgain | NM_145862 | c.C79T | p.Q27X |
| 1321 | 41 | Ⅰ | EC | *CHEK2* | missense mutation | NM_001257387 | c.G198T | p.K66N |
| 237 | 86 | Ⅲ | CCC | *EMSY* | missense mutation | NM_001300943 | c.G3880A | p.D1294N |
| 1027 | 37 | Ⅱ | CCC | *EMSY* | missense mutation | NM_001300943 | c.C3334T | p.R1112C |
| 1333 | 72 | Ⅲ | CCC | *EMSY* | missense mutation | NM_020193 | c.G319A | p.V107I |
| 1500 | 63 | Ⅲ | HGSC | *EMSY* | missense mutation | NM_001300943 | c.G3301A | p.G1101R |
| 756 | 74 | Ⅰ | CCC | *FANCL* | stopgain | NM_001114636 | c.G274T | p.E92X |
| 1030 | 47 | Ⅰ | CCC | *FANCL* | missense mutation | NM_001114636 | c.C62T | p.S21L |
| 1146 | 50 | Ⅰ | EC | *FANCL* | missense mutation | NM_001114636 | c.A554G | p.Q185R |

**Supplementary Table S2. Continued.**

| Patient ID | Age | Stage | Histological subtype | Gene | Class | Refseq ID | Nucleotide change | Amino acid change |
| --- | --- | --- | --- | --- | --- | --- | --- | --- |
| 1340 | 45 | Ⅲ | CCC | *FANCL* | missense mutation | NM_001114636 | c.C550T | p.P184S |
| 756 | 74 | Ⅰ | CCC | *FANCL* | splicing | NM_001114636 | c.274-1G>A |  |
| 109 | 54 | Ⅰ | EC | *NBN* | missense mutation | NM_002485 | c.C1670T | p.A557V |
| 246 | 57 | Ⅲ | HGSC | *NBN* | stopgain | NM_002485 | c.G2182T | p.E728X |
| 646 | 46 | Ⅰ | EC | *NBN* | stopgain | NM_002485 | c.G1147T | p.E383X |
| 1027 | 37 | Ⅱ | CCC | *NBN* | missense mutation | NM_002485 | c.C137T | p.A46V |
| 358 | 38 | Ⅰ | CCC | *PALB2* | missense mutation | NM_024675 | c.C757G | p.L253V |
| 762 | 38 | Ⅰ | EC | *PALB2* | missense mutation | NM_024675 | c.G458T | p.R153M |
| 1480 | 60 | Ⅰ | CCC | *PALB2* | missense mutation | NM_024675 | c.G2611C | p.D871H |
| 646 | 46 | Ⅰ | EC | *RAD50* | stopgain | NM_005732 | c.G1756T | p.E586X |
| 765 | 51 | Ⅰ | CCC | *RAD50* | missense mutation | NM_005732 | c.T3332C | p.M1111T |
| 1321 | 41 | Ⅰ | EC | *RAD51B* | missense mutation | NM_133510 | c.G529T | p.V177F |
| 1027 | 37 | Ⅱ | CCC | *RAD54L* | missense mutation | NM_003579 | c.C1046T | p.T349I |
| 1027 | 37 | Ⅱ | CCC | *RAD54L* | missense mutation | NM_003579 | c.A2030G | p.D677G |

**Supplementary Table S3. Clinical characteristics stratified by HR-associated gene mutation status in HGSC, CCC and EC**

| Histology | HGSC | |  |  | CCC | |  |  | EC | |  |
| --- | --- | --- | --- | --- | --- | --- | --- | --- | --- | --- | --- |
| Germline and/or somatic HR-associated gene mutation | + | - |  |  | + | - |  |  | + | - |  |
| Number of patients | 21 | 29 |  |  | 27 | 72 |  |  | 9 | 30 |  |
| Median Age (range) | 59 (46-81) | 61 (38-84) | p = 0.75 |  | 52 (37-86) | 55 (35-82) | p = 0.62 |  | 46 (38-54) | 53.5 (35-78) | p = 0.00061 |
| Stage* |  | | | | | | | | | | |
| I | 3 (14.3) | 4 (13.8) | p = 0.53 |  | 16 (59.3) | 45 (62.5) | p = 0.31 |  | 9 (100) | 19 (63.3) | p = 0.30 |
| II | 5 (23.8) | 4 (13.8) |  |  | 3 (11.1) | 9 (12.5) |  |  | 0 (0) | 5 (16.7) |  |
| III | 12 (57.1) | 17 (58.6) |  |  | 8 (29.6) | 12 (16.7) |  |  | 0 (0) | 4 (13.3) |  |
| IV | 1 (4.8) | 4 (13.8) |  |  | 0 (0) | 6 (8.3) |  |  | 0 (0) | 2 (6.7) |  |

*The number of parenthesis in Stage information is percentage.

**Supplementary Figure S1. The details of HR-associated gene mutations** **by clinical stage per histological subtype**


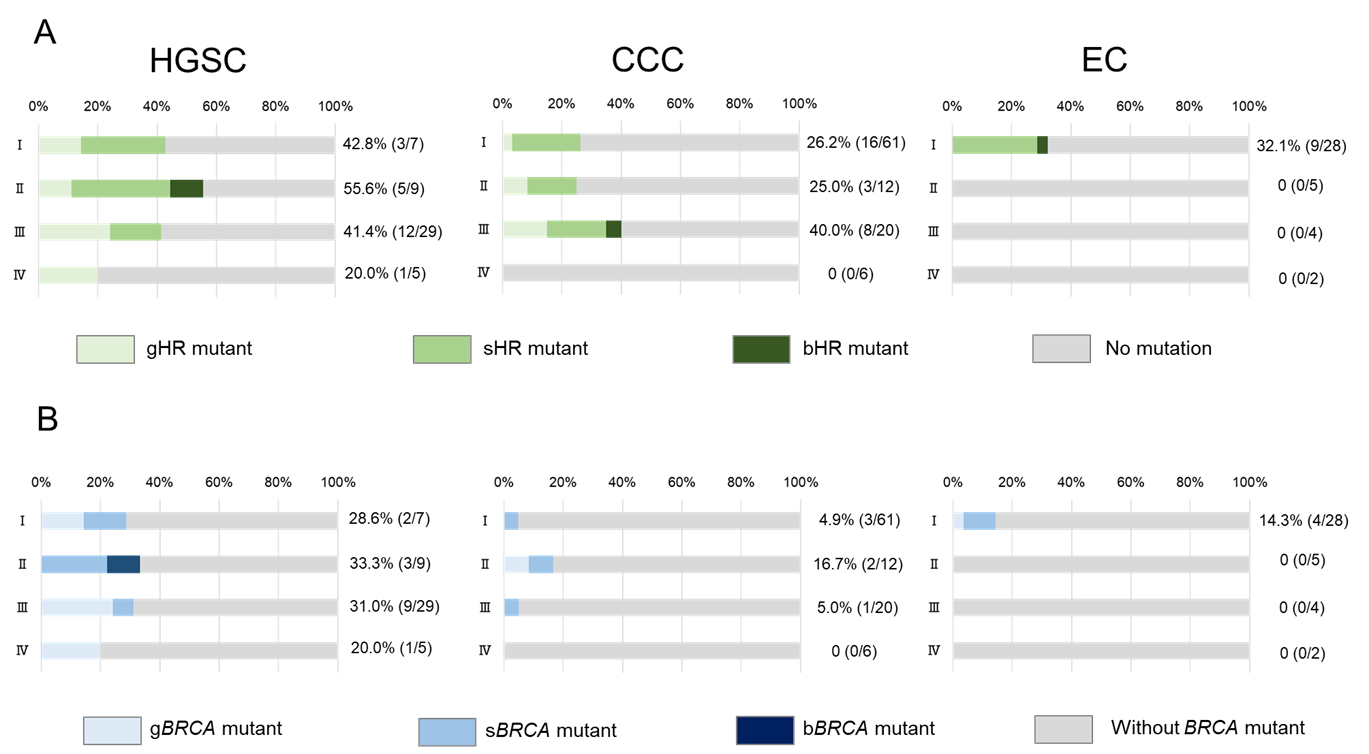


The frequency of HR-associated gene mutation and BRCA mutation by clinical stage per histological subtype is shown in 100 percent stacked bar chart. The mutation data were classified into four categories, respectively – (A) germline HR-associated gene mutation (gHR m), somatic HR-associated gene mutation (sHR m), both germline and somatic HR-associated gene mutation (bHR m) and no mutation (B) germline BRCA mutation (gBRCA m), somatic BRCA mutation (sBRCA m), both germline and somatic BRCA mutation (bBRCA m), and without BRCA mutation.

**Supplementary Figure S2. Association between *BRCA* mutations and clinical outcome per histological subtype**


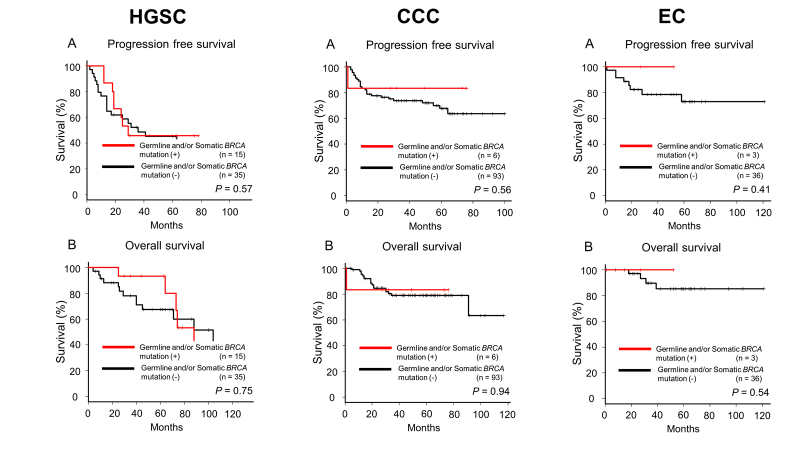


Kaplan–Meier estimates of progression-free survival (A) and overall survival (B) in HGSC, CCC, and EC.

**Supplementary Figure S3.** **Association between MMR gene mutations and HR-associated gene mutations**


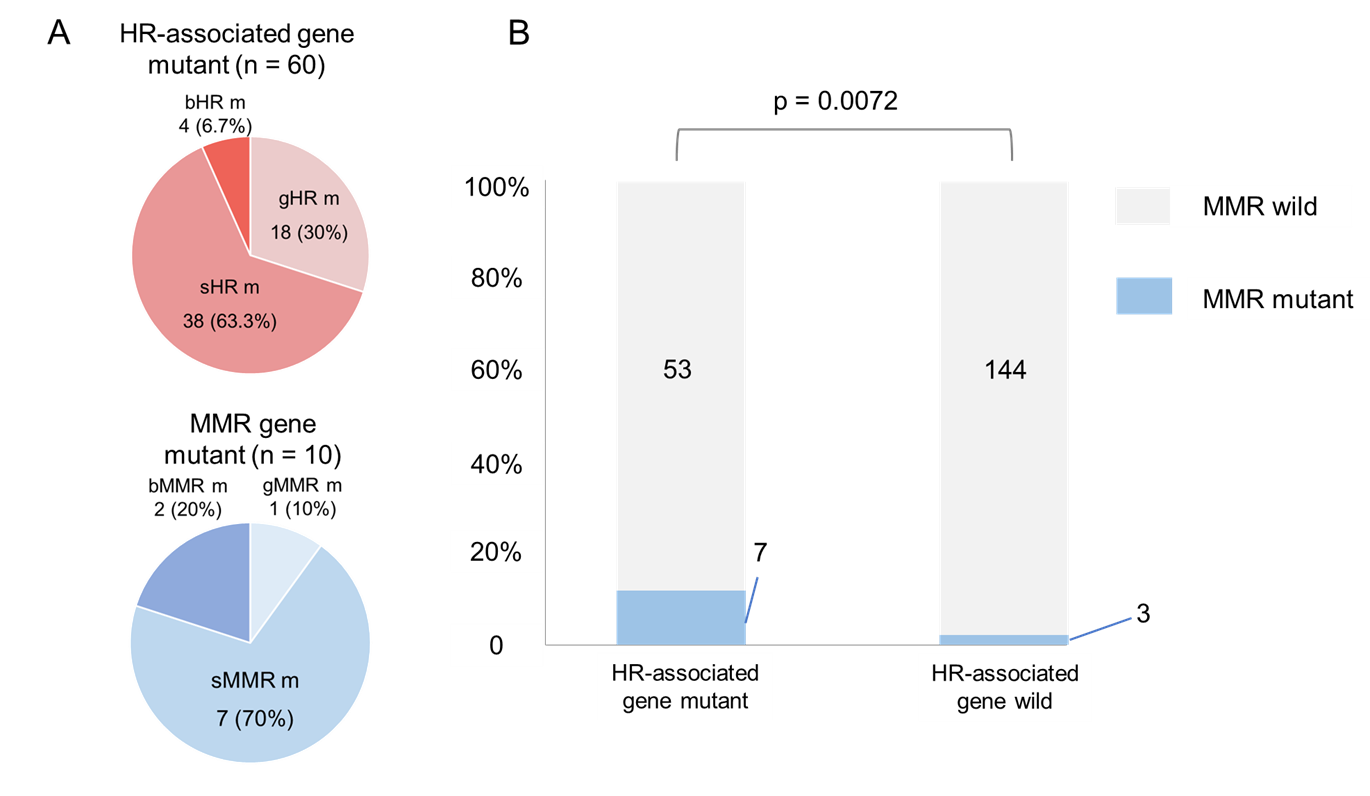


(A) The details of HR-associated gene mutation and MMR mutation are shown in pie chart. The mutation data were

classified into six categories –germline HR-associated gene mutation (gHR m), somatic HR-associated gene mutation (sHR m), both germline and somatic HR-associated gene mutation (bHR m), germline MMR mutation (gMMR m), somatic MMR mutation (sMMR m), and both germline and somatic MMR mutation (bMMR m). (B) Association between MMR gene mutations and HR-associated gene mutations is shown in 100% stacked column chart.
